# EUPopLink Country report - Montenegro

**DOI:** 10.12688/openreseurope.23527.1

**Published:** 2026-05-07

**Authors:** Andrijana Lazarevic, Stefan Surlić

**Affiliations:** 1 Political Science, University of Belgrade, Faculty of Political Sciences, Beograd, 11000, Serbia; 2 Political Science, University of Belgrade, Faculty of Political Sciences, Beograd, 11000, Serbia

**Keywords:** Montenegro, populism, Euroscepticism

## Abstract

This country report examines the relationship between populism and Euroscepticism in Montenegro within the framework of the EUPopLink COST Action. It situates Montenegro as a frontrunner in the EU accession process among Western Balkan countries, characterized by sustained pro-European consensus but increasing political fragmentation and institutional instability. The report identifies key populist actors, including the New Serb Democracy (NSD), Democratic People’s Party (DNP), Democratic Montenegro (Demokrate), United Reform Action (URA), Europe Now! (PES), and the Democratic Party of Socialists (DPS), and analyses their ideological profiles and positions toward the European Union.

The findings indicate that Euroscepticism in Montenegro is predominantly soft, instrumental, and discursive rather than ideological or programmatic. While identity-based narratives are evident among right-wing actors such as NSD and DNP, other parties employ valence or technocratic populism, using the EU as a benchmark for governance reform or as a rhetorical tool in domestic political competition. Despite growing criticism of EU conditionality and perceived inconsistency, no major political actor advocates withdrawal from the EU integration process.

The report further demonstrates that responses to Euroscepticism rely on a combination of institutional reforms, particularly in the rule of law, media governance, and anti-corruption measures, alongside strategic communication of EU benefits. Overall, populist Euroscepticism in Montenegro remains limited in its capacity to shape policy direction, functioning primarily as a mechanism of political contestation rather than a transformative force.

## Plain Language summary

This report examines how political parties in Montenegro position themselves toward the European Union (EU) and how populism shapes this dynamic. Montenegro has been advancing toward EU membership for many years, and most political actors continue to support this objective. However, the political landscape has become more polarized, and trust in institutions has declined.

Some parties use populist narratives to challenge political elites, corruption, or external influence. Others invoke the idea of Europe to present themselves as modern, reform-oriented, and effective. Even when parties express criticism toward the EU, they rarely oppose accession itself. Instead, they question the EU’s role, consistency, or level of engagement in supporting reforms in Montenegro.

Overall, Euroscepticism in Montenegro remains relatively mild and is primarily used as a political tool rather than a basis for policy change. The country’s EU trajectory remains stable, with most actors continuing to view the EU as essential for economic development, strengthening the rule of law, and improving governance.

## 1. Introduction

The present country report was prepared as part of the EUPopLink COST Action ^1^ following the EUPopLink Theoretical Framework and guidelines. ^2^

Montenegro has been on the path toward European Union (EU) integration since gaining independence in 2006, with official candidate status since 2010 and accession negotiations starting in 2012. Unlike in many EU member states and some of the Western Balkan candidate countries, European integration has long enjoyed a broad cross-party elite consensus and popular support. The EU has been viewed not only as a strategic foreign policy goal but as an anchor of political, legal, and economic modernization.

The country opened accession negotiations with the EU in 2012 and has since opened all 33 negotiating chapters, with seven provisionally closed. ^3, 4^ Since 2007, the European Union has provided approximately €1.2 billion in non-repayable grants directly to Montenegro, making it the largest foreign donor. ^5^ Montenegro has also benefited from EU-supported regional programs aimed at socio-economic development and institutional reform. ^6^

Although it formally leads the Western Balkans in alignment with EU standards, reform momentum has slowed due to political instability, institutional crises, and a polarized landscape. Public support for EU membership remains relatively high but fluctuates over time rather than declining. According to the 2024 Balkan Barometer, 51 percent of citizens believe that EU membership would be good for the country, while about 40 percent are neutral or negative. ^7, 8^ In a referendum scenario, 64 percent would vote in favour of accession, although only 39 percent expect membership by 2030. Citizens highlight three priority areas for a stronger EU role: economic development (48%), anti-corruption measures (45%), and support for local projects in infrastructure, agriculture, health, environment, and culture (40%). ^9^ These trends show that while enthusiasm varies slightly, overall support for EU membership in Montenegro remains relatively high when associated with tangible governance and economic benefits. ^10^

Despite this pro-European orientation, the post-2008 period witnessed the emergence of Eurosceptic discourses, largely driven by political polarization, the erosion of trust in domestic elites, and identity politics. While populism in Montenegro is not as deeply entrenched as elsewhere, it is increasingly invoked in elite competition and identity-based mobilization targeting EU influence.

## 2. Populist political actors

Since independence, a distinct form of state-sponsored populism has emerged, driven by ruling elites rather than ideological outsiders. As Keil and Džankić ^11^ (2017) note, this elite-controlled populism maintains clientelist networks through moral and identity narratives while cloaking itself in pro-European rhetoric. This dynamic intensified during the 2019–2020 protests against the Law on Religious Freedoms, when political actors mobilized around religious identity and traditional values. ^12^

Following the June 2023 election, the seat distribution was as follows: Europe Now! (PES) 24 seats; Democratic Party of Socialists (DPS) 21; “For the Future of Montenegro” (ZbCG – including NSD and DNP) 13; Democrats (DCG) and United Reform Action (URA) 11; Bosniak Party (BS) 6; SNP-DEMOS 2; minority lists 4. ^13, 14^

### New serb democracy (Nova srpska demokratija – NSD) ^15^

Led by Andrija Mandić, NSD represents the clearest example of populist radical right (PRR) politics in Montenegro. Its discourse is built around Serb ethno-national identity, Orthodox religious values, and opposition to Western liberalism, framing politics as a battle between the “authentic Serbian people” and a corrupt Westernised elite. As part of the ZbCG bloc, NSD and its allies won 13 seats in 2023; Mandić currently serves as Speaker of Parliament. The party has no formal European affiliation but maintains informal ties with Serbia’s SNS and regional right-wing networks.

### Democratic people’s party (Demokratska narodna partija – DNP) ^16^

Led by Milan Knežević, the DNP shares much of NSD’s clerical-nationalist orientation but adopts a more confrontational sovereigntist tone and hardline anti-elitism. Also within the ZbCG bloc (13 seats total), DNP is ideologically close to NSD and similarly outside any European party family. ^13^

### Democratic montenegro (Demokratska Crna Gora – Demokrate) ^17^

Under the leadership of Aleksa Bečić, the Demokrate embody valence populism focused on anti-corruption, moral renewal. They avoid radical nationalism and present a “clean hands” alternative to the old elite. Through the ‘Bravery Counts!’ coalition with URA (11 seats in 2023), they position themselves as a civic, centre-right alternative. They cooperate with EPP circles through PACE fora but lack formal EPP membership.

### United reform action (Građanski pokret URA) ^18^

Led by Dritan Abazović, URA positions itself as a green-liberal and civic reformist party using anti-establishment populism rhetoric to oppose state capture and corruption. URA’s core message revolves around the “fight against the captured state” positioning itself as the civic antidote to a corrupt duopoly of DPS and traditional opposition. In 2023 URA joined the Demokrate bloc (11 seats total) and since December 2022 has been a full member of the European Green Party. ^19^

### Europe now! (Pokret Evropa sad!) ^20^

Co-founded by Milojko Spajić and Jakov Milatović, PES represents the newest political force in Montenegro (since 2022), a technocratic populist movement that rose rapidly to become the largest parliamentary force with 24 seats ^14^ (PolitPro, 2023). It frames politics around economic efficiency and modernisation, contrasting its “delivery model” with old elite inertia. Although ideologically ambiguous, PES is strongly pro-European and forms the core of the current government.

### Democratic party of socialists (Demokratska partija socijalista – DPS) ^21^

The long-ruling DPS of Milo Đukanović held power for three decades and remains a major player with 21 seats (2023). It historically combined pro-European and state-sponsored populism. It cast itself as the defender of Montenegrin independence, multiculturalism, and EU integration, contrasting this with “backward” nationalist forces. The Democratic Party of Socialists has been a full member of the Socialist International since 2008, and an associate member of the Party of European Socialists since 2009, aligning with social-democratic networks. ^22^

## 3. Positions on ‘Europe’ and the populism-Euroscepticism nexus

The main populist actors shaping the European integration debate include NSD, DNP, Demokrate, URA, and PES, with DPS remaining the primary pro-EU reference point. Minor parties show sporadic Euroscepticism but minimal electoral impact.

Unlike the ideological Euroscepticism found in some EU member states, Montenegrin Euroscepticism is soft and instrumental – used to criticise elites and EU conditionality rather than to reject integration ^11^ (Keil & Džankić, 2017; ISS, 2024).

*New Serb Democracy* promotes *identity-based soft Euroscepticism,* portraying the EU as liberal and morally distant, allegedly undermining the Serbian Orthodox Church and traditional values. It does not explicitly oppose EU accession but is deeply skeptical of the EU’s liberal normative agenda. Brussels is framed as complicit in supporting a corrupt, pro-Western elite and undermining the Serbian Orthodox Church and Serb identity. The party’s clerical-nationalist discourse often includes references to traditional values and sovereignty, occasionally echoing pro-Russian and pan-Serbian sentiments. While critical of EU influence, NSD’s opposition is more cultural and normative than institutional, expressing concern over external liberalism rather than advocating for withdrawal from the European path.

*Democratic People’s Party* articulates a more confrontational sovereigntist variant, portraying the EU as biased and elitist and anti-traditional, drawing on regional right-wing frames. The party denounces Brussels for enabling a captured state and tolerating illiberalism when aligned with its strategic interests. DNP positions itself as a defender of national and religious values and borrows rhetorical cues from regional right-wing populist movements. While it lacks formal European affiliations, DNP draws rhetorical inspiration from right-wing populist movements in the region.

*The Demokrate* maintain formal Europhilia but emphasise the EU’s “passivity” toward Montenegrin corruption - reflecting *conditional, instrumental support* rather than ideological skepticism. ^23^ The party avoids explicit ideological Euroscepticism and does not question the EU’s role or future; instead, it presents Europe as a normative benchmark inconsistently applied. Their framing of “honest citizens vs. a rotten political caste” includes EU actors only insofar as they are seen as tolerating domestic misgovernance. As such, the Demokrate express a conditional and instrumental commitment to the EU project.

*URA links* EU to institutional renewal and civic values, criticising Brussels for insufficient engagement and it often frames Brussels as too passive in addressing local democratic backsliding. The party positions the EU as both an aspirational goal and a tool for dismantling the captured state. Criticism of the EU emerges when it is seen as disengaged or complicit in democratic stagnation, particularly regarding anti-corruption and judiciary reform. This critique is instrumental rather than ideological, as URA consistently frames Europe as a normative ally. Its populism lies in its claim to be the sole authentic civic force capable of restoring European values internally positioning Brussels as a neglected but still legitimate partner in institutional renewal.

*Europe Now!* represents a technocratic populism that is outwardly pro-European yet strategically critical; but simultaneously expresses dissatisfaction with Brussels’ failure to address Montenegro’s democratic and economic inertia; uses the EU as a symbol of efficiency and modernisation while attacking old corrupt structures in the country. ^20^ Its discourse emphasizes depoliticized governance, efficiency, and public sector reform, casting its leadership as competent outsiders who will deliver “European standards” without the baggage of political elites. While avoiding traditional Eurosceptic tropes, PES uses the idea of Europe rhetorically - to contrast its vision of pragmatic transformation with the status quo, rather than to question EU membership itself.

*Democratic Party of Socialists*, as the long-time ruling party, DPS crafted Montenegro’s pro-EU narrative, presenting European integration as synonymous with sovereignty, modernization, and post-independence legitimacy. However, its European credentials were gradually undermined by authoritarian tendencies, state capture, and systemic corruption. Despite its rhetorical Europhilia and alignment with the Party of European Socialists (PES), the party’s governance practices led to widespread disillusionment and accusations of instrumentalizing the EU for regime survival. This disjuncture between discourse and practice helped erode public trust in pro-European messaging and opened space for new populist actors to redefine Montenegro’s European orientation. While not Eurosceptic, DPS exemplifies a case where nominal pro-Europeanism masked illiberal governance and weakened the normative appeal of EU integration.

Overall, Euroscepticism in Montenegro is predominantly soft and instrumental, identity-inflected among NSD/DNP, valence and anti-corruption among Demokrate, and civic-technocracy within URA and PES. No major party advocates withdrawal from the EU while the cleavage remains discursive rather than strategic. ^13, 14^

## 4. Responses to Euroscepticism and consequences

Because of Montenegro’s volatile party system, responses to Euroscepticism are fragmented. Historically, DPS acted as the dominant pro-EU force, branding opponents as backward or foreign-influenced. Its rejection of the “Open Balkan” initiative illustrates this stance – the government framed the project as Serbia-dominated and potentially undermining the EU-led Berlin Process. Unlike North Macedonia and Albania, Montenegro argued that the Berlin Process and CEFTA already provided adequate frameworks. ^24, 25^ This position reflected a “Berlin-Process-first” approach rather than isolationism.

By 2020, DPS lost credibility over corruption and state capture, allowing new actors to appropriate the European narrative through “clean-governance” frames (PES, URA) or identity-based critique (NSD, DNP). Smaller civic actors like URA remained pro-European but criticised EU leniency toward DPS, marking a shift from normative alignment to pragmatic critique.

Institutionally, Euroscepticism has had limited impact: no major party seeks to reverse accession. The 2023 parliamentary composition (PES 24, DPS 21, ZbCG 13, DCG/URA 11, BS 6) confirms that even critical actors remain within a pro-EU framework.

Rule-of-law hardening under Chapters 23/24 has been central. Updated action plans introduced measurable benchmarks for judicial independence and anti-corruption performance, while the positive IBAR review marked a turning point for negotiation credibility. ^6, 3^

Media governance and disinformation resilience were strengthened through the 2020 *Law on Media* and *Law on the Public Broadcaster*, followed by 2024–25 alignment with the EU AVMS Directive, transparency of ownership, and empowerment of the regulator. ^26, 27^ Parallel literacy and fact-checking initiatives by the Digital Forensic Center and AEM mitigate identity-based and foreign-influenced populism. ^28^

The dominant antidote to populism and Euroscepticism combines institutional delivery (Chapters 23/24), media resilience, and targeted EU communication. While identity-based frames (NSD/DNP) persist, the political cost of an explicitly anti-EU stance has risen as reforms advance, and citizens perceive concrete benefits through local projects and the EU Growth Plan. ^6, 29^ Hence, the most effective response to populism and Euroscepticism in Montenegro is a blend of deliverology, media resilience, and consensus messaging rather than pure rhetorical confrontation between parties.

Finally, external factors, such as the war in Ukraine or the EU’s renewed enlargement discourse, have not significantly shifted party positions in Montenegro. Rather than triggering deep ideational change, such events tend to be refracted through domestic political agendas. For instance, while pro-Serb parties have echoed Russian talking points in the context of Ukraine, this has not translated into explicit anti-EU policy proposals. Similarly, pro-European actors continue to stress alignment with Brussels while questioning the EU’s credibility or consistency.

## 5. Conclusion

Montenegro’s populism and Euroscepticism remain soft, instrumental, and non-programmatic. While no major party openly rejects the goal of EU accession, several populist actors express varying degrees of soft or instrumental Euroscepticism, often rooted in identity politics, frustration with domestic elites, or criticism of EU engagement practices. This Euroscepticism is rarely ideological or programmatic; rather, it serves as a tool for political differentiation and electoral mobilization. Although populist discourse has reshaped how ‘Europe’ is framed in public debate, it has not significantly altered Montenegro’s official trajectory toward EU membership. Much like in other cases, a gap persists between populist rhetoric and concrete policy, suggesting that Euroscepticism in Montenegro functions more as a flexible narrative than a fixed strategic objective. Its limited institutional impact suggests that populist Euroscepticism in Montenegro is more discursive than transformative, serving as a vehicle for domestic contestation rather than a coherent critique of the EU.

## Ethics and consent

Ethical approval and consent were not required.

## Data Availability

No new data were generated or analysed in this manuscript. All data are derived from publicly available sources, which are cited in the reference list.
