# Supplementary material for: EUPopLink Country report - Montenegro
Source: Open Res Eur. 2026 May 7;6:152. [Version 1] doi: 10.12688/openreseurope.23527.1 (PMC13324361; doi:10.12688/openreseurope.23527.1)
Supplement: Peer review history

# Reviewer report: Reviewer response for version 1

**DOI:** 10.21956/openreseurope.25470.r76375

**Published:** 2026-07-01

**Authors:** Vit Hlousek

**Affiliations:** 1 Metropolitan University Prague, Prague, Czech Republic

The paper represents an attempt of synthetizing the current state of Euroscepticism in Montenegro. It is based on a solid knowledge of the topic, and the authors tackle all important issues related to the topic. They do it in an organized and basically accessible way. Yet I would love to encourage them to make some changes and amendments in organizing their material.

 First of all, a brief delimitation of their treatment of the concepts like Euroscepticism or soft Euroscepticism would be welcomed. A brief definition complemented with the link to the literature would do the job well enough.

 Second, a reader who is not familiar with the Montenegrin party system deserves a more detailed and coherent description of it, including to pro-integration parties. The sound descriptive paragraph, placed ideally to the intro of part 2, is a must for anyone who is not a country expert on Montenegro.

 Third, the section 3 shall be reorganized. Now it reads almost like a bullet-styled set of brief singular descriptions of the particular Eurosceptic actors. A better organized analysis, grouping the parties according to the nature of their Euroscepticism and populism would serve its purpose better.

Is the case presented with sufficient detail to be useful for teaching or other practitioners?

Partly

Is the work clearly and accurately presented and does it cite the current literature?

Partly

If applicable, is the statistical analysis and its interpretation appropriate?

Not applicable

Are all the source data underlying the results available to ensure full reproducibility?

No source data required

Are the conclusions drawn adequately supported by the results?

Yes

Is the background of the case’s history and progression described in sufficient detail?

Partly

Reviewer Expertise:

comparative politics, European governance, East-Central European studies, contemporary history

I confirm that I have read this submission and believe that I have an appropriate level of expertise to confirm that it is of an acceptable scientific standard, however I have significant reservations, as outlined above.

# Reviewer report: Reviewer response for version 1

**DOI:** 10.21956/openreseurope.25470.r76371

**Published:** 2026-06-29

**Authors:** Sandro Tabatadze

**Affiliations:** 1 Tbilisi State University, Tbilisi, Tbilisi, Georgia

The report addresses a relevant and analytically interesting case, provides useful coverage of the main political actors, and offers a promising basis for comparative analysis.

 Here are some suggestions for further improvement: 

 1. The last part of the introduction ("While populism in Montenegro is not as deeply entrenched as elsewhere, it is increasingly invoked in elite competition and identity-based mobilization targeting EU influence") may raise further questions. The authors should clarify this argument in an additional 1–2 sentences.

 2. The second paragraph of the second part seems out of context. It may be difficult for readers to understand the connection between the parties listed here and those discussed separately later. It is worth noting that the following section addresses both populist parties that have entered parliament and extra-parliamentary populist actors.

 3.It would enhance clarity to group the parties presented in Part 2 by criteria such as the type of populist message they convey (for example, the first group could include radical right-wing populists, while the second could focus on valence populists). This organisation would offer readers a more comprehensive picture of populist party actors rather than a fragmented one. Moreover, it may be challenging for readers to differentiate between opposition and government parties. Clearly indicating this in the chapter would provide a more structured framework for the report.

 4. The cases discussed in Part 3 are well-analyzed; however, the presence of many different actors may lead to a loss of systematic coherence in the report. Once again, it would be better to present these six parties in relation to the nexus between Euroscepticism and populism.

 5. The report should also clarify whose (domestic political parties, state institutions, civil society, the EU, or the broader accession framework) responses are being analysed in Part 4. Currently, these levels are mixed. It is essential to distinguish among party-based, institutional, and EU-level responses.

 6. The report also needs to be more cautious about its claims of effectiveness (Hence, the most effective response to populism and Euroscepticism in Montenegro is...).Claims that media resilience, targeted EU communication, or the EU Growth Plan mitigate Euroscepticism should be supported by evidence or presented more carefully as plausible mechanisms rather than established findings.

 7. A conclusion should highlight that Montenegro is a case in which populist actors use EU-related discourse for domestic competition, while the goal of EU accession remains the primary strategic objective. This positions Montenegro differently from other cases.

 8. As Montenegro is an EU candidate country, it would have been better to draw on existing literature on Euroscepticism and populism in EU candidate states, particularly since the paper centres on EU conditionality.

Is the case presented with sufficient detail to be useful for teaching or other practitioners?

Yes

Is the work clearly and accurately presented and does it cite the current literature?

Yes

If applicable, is the statistical analysis and its interpretation appropriate?

Not applicable

Are all the source data underlying the results available to ensure full reproducibility?

Yes

Are the conclusions drawn adequately supported by the results?

Yes

Is the background of the case’s history and progression described in sufficient detail?

Yes

Reviewer Expertise:

Euroscepticism, populism, EU candidate states, Far right.

I confirm that I have read this submission and believe that I have an appropriate level of expertise to confirm that it is of an acceptable scientific standard, however I have significant reservations, as outlined above.

# Reviewer report: Reviewer response for version 1

**DOI:** 10.21956/openreseurope.25470.r75317

**Published:** 2026-06-26

**Authors:** Adriana Ștefănel

**Affiliations:** 1 Faculty of Journalism and Communication Studies, Universitatea din Bucuresti, Bucharest, Bucharest, Romania

This is a well-structured and informative country report that provides a useful overview of the relationship between populism and Euroscepticism in Montenegro. The manuscript is clearly written, logically organized, and offers a valuable contribution to the comparative objectives of the EUPopLink project. The authors successfully demonstrate that Euroscepticism in Montenegro remains predominantly soft, instrumental, and embedded within a broader pro-European consensus.

 The reason several evaluation criteria were assessed as ***Partly*** rather than ***Yes*** is not because of factual inaccuracies, but because some analytical claims would benefit from further elaboration and stronger justification.

1. Regarding the **background and historical development**, the report provides sufficient context on Montenegro's EU accession process and contemporary political landscape. However, the historical evolution of populist and Eurosceptic narratives could be developed further, particularly concerning the transition from the long period of DPS dominance to the fragmented post-2020 political environment.
2. The manuscript is generally well grounded in the literature, but some conceptual classifications require additional justification. In particular, the classification of **Europe Now! (PES)** as a form of *technocratic populism* is plausible but not self-evident. The report would benefit from a clearer explanation of which characteristics justify its inclusion among populist actors and how this classification relates to the broader literature on technocratic and populist movements.
3. Similarly, the characterization of **DPS as a form of "state-sponsored populism"** relies on existing scholarship but remains a relatively strong and potentially controversial interpretation. A brief discussion of the criteria used for this classification would strengthen the analytical transparency of the report.
4. The report contains limited original quantitative analysis and relies primarily on descriptive statistics and secondary sources. This is entirely appropriate for a country report format, but some broader conclusions extend beyond the empirical evidence presented and therefore cannot be considered fully demonstrated.

I would also encourage the authors to reconsider the statement that ***external factors have not significantly shifted party positions in Montenegro*** ***.*** While the overall pro-EU consensus has remained stable, developments such as Russia's invasion of Ukraine, changes in the EU enlargement agenda, and evolving geopolitical alignments have influenced political discourse and foreign policy positioning. A more nuanced formulation would likely be preferable.

 Finally, the report would become more useful for teaching and practitioner audiences if it included a few concrete examples of political discourse, campaign messages, or public controversies illustrating how Eurosceptic narratives are mobilized in practice.

 Overall, the manuscript is informative, well documented, and makes a valuable contribution to understanding the Montenegrin case. The revisions suggested above are relatively minor and are intended primarily to strengthen conceptual clarity and analytical precision.

Is the case presented with sufficient detail to be useful for teaching or other practitioners?

Partly

Is the work clearly and accurately presented and does it cite the current literature?

Partly

If applicable, is the statistical analysis and its interpretation appropriate?

Partly

Are all the source data underlying the results available to ensure full reproducibility?

Partly

Are the conclusions drawn adequately supported by the results?

Partly

Is the background of the case’s history and progression described in sufficient detail?

Partly

Reviewer Expertise:

My research expertise lies primarily in political communication, media studies, political discourse, populism, Euroscepticism, digital media, social media communication, political behavior, public opinion, and research methodology. I have particular experience in the analysis of political narratives, populist communication, European integration debates, media framing, and comparative studies of political actors and public discourse.

I confirm that I have read this submission and believe that I have an appropriate level of expertise to confirm that it is of an acceptable scientific standard.
